# Spatial CT perfusion data helpful in automatically locating vessel occlusions for acute ischemic stroke patients

**DOI:** 10.3389/fneur.2023.1136232

**Published:** 2023-03-29

**Authors:** Daan Peerlings, Hugo W. A. M. de Jong, Edwin Bennink, Jan W. Dankbaar, Birgitta K. Velthuis, Bart J. Emmer, Charles B. L. M. Majoie, Henk A. Marquering

**Affiliations:** ^1^Department of Radiology, University Medical Center Utrecht, Utrecht, Netherlands; ^2^Image Sciences Institute, University Medical Center Utrecht, Utrecht, Netherlands; ^3^Department of Radiology and Nuclear Medicine, Amsterdam University Medical Centers, Location Academic Medical Center, Amsterdam, Netherlands; ^4^Department of Biomedical Engineering and Physics, Amsterdam University Medical Centers, Location Academic Medical Center, Amsterdam, Netherlands

**Keywords:** brain ischemia, cerebrovascular occlusions, perfusion imaging, stroke, tomography, X-ray computed

## Abstract

**Introduction:**

Locating a vessel occlusion is important for clinical decision support in stroke healthcare. The advent of endovascular thrombectomy beyond proximal large vessel occlusions spurs alternative approaches to locate vessel occlusions. We explore whether CT perfusion (CTP) data can help to automatically locate vessel occlusions.

**Methods:**

We composed an atlas with the downstream regions of particular vessel segments. Occlusion of these segments should result in the hypoperfusion of the corresponding downstream region. We differentiated between seven-vessel occlusion locations (ICA, proximal M1, distal M1, M2, M3, ACA, and posterior circulation). We included 596 patients from the DUtch acute STroke (DUST) multicenter study. Each patient CTP data set was processed with perfusion software to determine the hypoperfused region. The downstream region with the highest overlap with the hypoperfused region was considered to indicate the vessel occlusion location. We assessed the indications from CTP against expert annotations from CTA.

**Results:**

Our atlas-based model had a mean accuracy of 86% and could achieve substantial agreement with the annotations from CTA according to Cohen's kappa coefficient (up to 0.68). In particular, anterior large vessel occlusions and occlusions in the posterior circulation could be located with an accuracy of 80 and 92%, respectively.

**Conclusion:**

The spatial layout of the hypoperfused region can help to automatically indicate the vessel occlusion location for acute ischemic stroke patients. However, variations in vessel architecture between patients seemed to limit the capacity of CTP data to distinguish between vessel occlusion locations more accurately.

## Introduction

The location of a vessel occlusion is central to the clinical decision support of acute ischemic stroke patients. To diagnose these patients, multimodal CT imaging is performed, consisting of non-contrast CT, CT angiography (CTA), and CT perfusion (CTP). Non-contrast CT can be used to exclude hemorrhagic stroke and stroke mimics, after which CTP can be used to distinguish the infarcted tissue from the salvageable tissue, and CTA can be used to locate the vessel occlusion (1).

Mechanical thrombectomy has become the standard of care for patients suffering from acute ischemic stroke due to anterior circulation proximal large vessel occlusions (2). Recent advancements in stent retriever technology and thromboaspiration devices may widen the eligibility criteria for endovascular treatment by considering occlusions in smaller and more distal vessels (3). It is a prerequisite for endovascular treatment to locate vessel occlusion.

Reading CTA to locate vessel occlusions can be challenging. The more distal the vessel occlusion, the harder this task becomes because the occlusion is smaller, has delayed opacification, can be concealed behind cortical veins, and can be found in a larger number of vessels that exhibit more anatomical variability (4, 5). Moreover, while less experienced readers are likely on call in the emergency setting, they do not perform as well as experienced readers in giving fast and accurate evaluations (6, 7). Therefore, the advent of endovascular treatment beyond proximal large vessel occlusions spurs alternative approaches to locate vessel occlusions (8).

CTP provides a way to locate vessel occlusions because a vessel occlusion results in a perfusion deficit in the downstream region of the occluded vessel (3, 5, 9, 10). As such, it has been shown that experienced readers of CTP can accurately discern patients with distal vessel occlusion from patients without vessel occlusion (5). Moreover, the addition of CTP to CTA has expedited and improved the detection of vessel occlusions on CTA in previous studies (9, 10). Hence, CTP data may contain information that is helpful in locating vessel occlusions.

CTP data on its own are often regarded as complex because it consists of several physiological parameters. Although the presence of a perfusion deficit suffices to detect a vessel occlusion, assessing this deficit to locate the vessel occlusion requires additional physiological and neuroanatomical expertise. Therefore, an automatic evaluation of CTP data may facilitate the support of CTP in reading CTA.

In this study, we explore the potential of CTP to automatically locate vessel occlusions. We present an atlas-based method in which CTP data are used to indicate the vessel occlusion location. These indicated vessel occlusion locations were compared with annotated vessel occlusion locations from expert readings of CTA.

## Methods

###  Acquiring imaging data from the patient population

Retrospective data were obtained from the DUtch acute STroke (DUST) study, in which 14 stroke centers in the Netherlands participated (11). All included DUST participants gave informed consent for the use of their imaging and clinical data. Patients that were admitted to the DUST study were suspected of an ischemic stroke and underwent non-contrast CT, CTP, and CTA imaging within 9 h after symptom onset. Only patients with both an annotated vessel occlusion location (obtained from CTA) and a perfusion deficit (obtained from CTP) were considered in this study.

The CTP protocol consisted of scanning at 80 kVp and 150 mAs on 40- to 320-detector CT scanners (GE Healthcare, Philips, Siemens, or Toshiba) with a 2 s interval for a duration of 50 s. The scans were reconstructed as 5 mm contiguous axial slices. The advised injection protocol was a 40 mL contrast bolus injected at a rate of 6 mL/s followed by a saline flush of 40 mL injected at a rate of 6 mL/s.

For the CTA, a 50–70 mL contrast bolus was injected at a rate of 6 mL/s followed by a saline flush of 40 mL injected at a rate of 6 mL/s. Centers could adhere to their own scanning protocol (such as setting the kVp and the mAs). The CTA scan delay after contrast injection was determined on a per-patient basis either from the time to peak arterial enhancement on CTP or by a trigger based on a threshold for the attenuation measured in the aortic arch during contrast enhancement.

The multicenter DUST data were processed centrally in the University Medical Center Utrecht. The CTA scans were examined with commercially available software on an Extended Brilliance Workstation (IntelliSpace Portal 4.5, Philips Healthcare). All further data processing and analysis were carried out with MATLAB (MATLAB, R2019b: The Mathworks Inc.).

###  Annotating vessel occlusion locations with CTA

**Figure 1:**
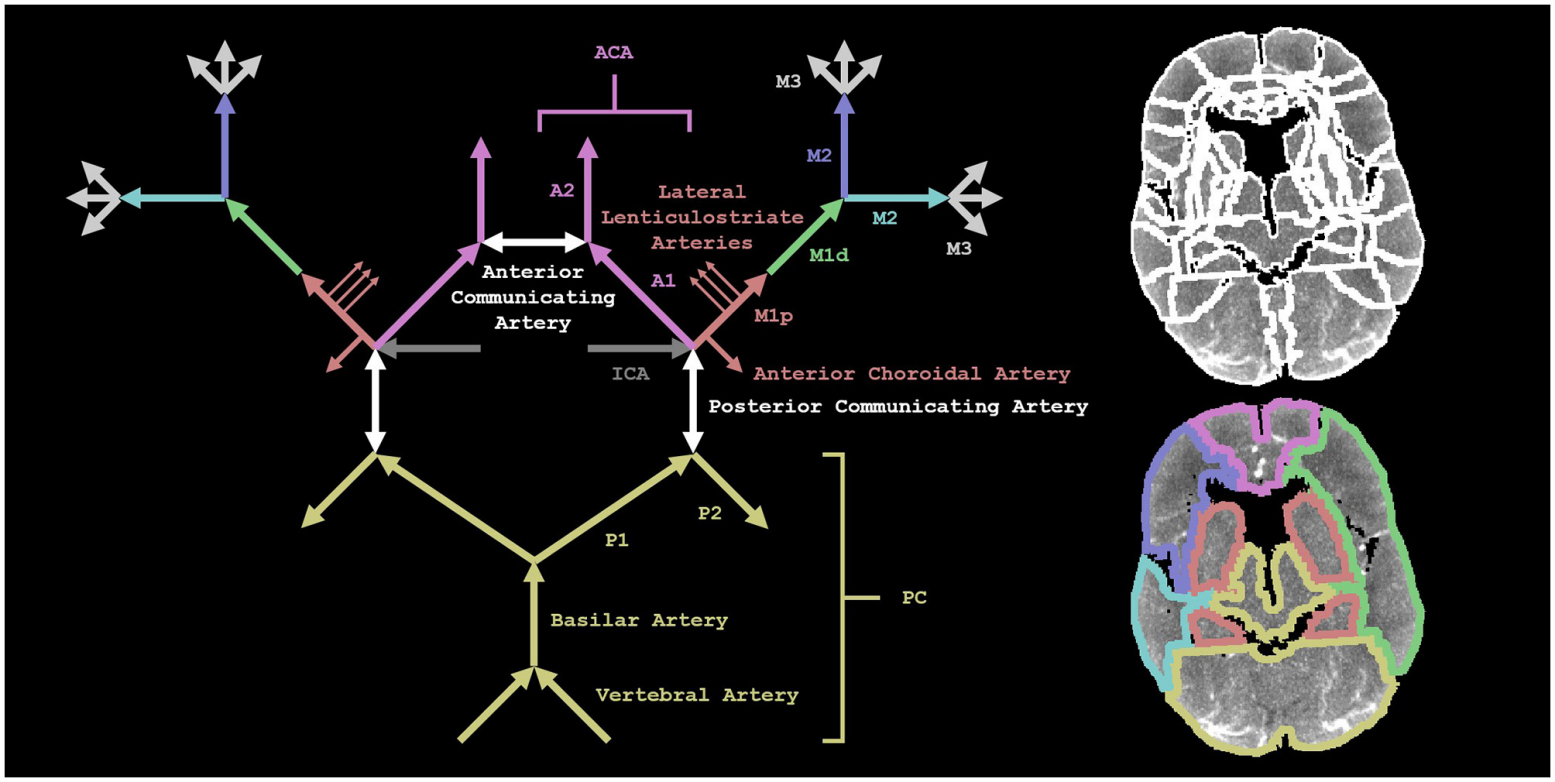
A visual representation of our methods. On the left is a schematic depiction of the arterial blood supply of the brain. The annotations from CTA did not specify between different M2 (or M3) branches. On the top right, we show the regions from a neuroanatomical CT-MRI brain atlas specific to the stroke population (registered to the CT perfusion scan of a patient) (12). On the bottom right, the neuroanatomical regions (shown on the top right) are grouped to constitute downstream regions that correspond to the vessel occlusion locations (shown on the left). We show the M1d downstream region in the left hemisphere and the two M2 downstream regions in the right hemisphere.

The vessel occlusion locations on CTA were defined as shown in Table 1 (also see Figure 1) and were termed ICA, M1p, M1d, M2 (anterior and posterior), M3, ACA, and PC. An expert reader (from a pool of three readers with at least 5 years of experience in neurovascular imaging) annotated the CTA images with the most proximal vessel occlusion location by reviewing thin slice CTA data with adjustable maximum intensity projection and multiplanar reformatting. The reader was blind to all clinical information other than the side of the symptoms. In Table 1, we differentiated between anterior M2 and posterior M2 occlusions because these have different downstream regions, but both were annotated as M2 on the CTA images.

**Table 1:** The definitions of the vessel occlusion locations and their downstream regions.

**Vessel occlusion location**	**Vasculature (CTA)**	**Downstream region (CTP)**
ICA	Up to the bifurcation of the internal carotid artery.	The collection of neuroanatomical regions from the M1p downstream region and either the ACA downstream region or the PC downstream region.
M1p (proximal M1)	From the bifurcation of the internal carotid artery up to the end of the lateral lenticulostriate arteries.	The collection of neuroanatomical regions from the M1d downstream region together with the neuroanatomical regions that are supplied by the anterior choroidal artery (entorhinal region, parahippocampus, hippocampus, and amygdala) and the neuroanatomical regions that are supplied by the lateral lenticulostriate arteries (caudate, pallidum, putamen, anterior limb of internal capsule, retrolenticular part of internal capsule, posterior limb of external capsule, and external capsule).
M1d (distal M1)	From the end of the lateral lenticulostriate arteries to the bifurcation of the middle cerebral artery.	The collection of neuroanatomical regions from the M2 downstream regions (anterior and posterior).
M2 (anterior)	From the bifurcation of the middle cerebral artery to where arteries exit the Sylvian fissure (the annotations from CTA did not specify between different M2 branches).	Rostral middle frontal region, caudal middle frontal region, pars orbitalis of inferior frontal region, pars opercularis of middle frontal region, pars triangularis of middle frontal region, precentral region, insular region, frontal white matter, anterior corona radiata, superior corona radiata, superior fronto-occipital fasciculus, and uncinate fasciculus.
M2 (posterior)	From the bifurcation of the middle cerebral artery to where arteries exit the Sylvian fissure (the annotations from CTA did not specify between different M2 branches).	Postcentral region, supramarginal region, superior parietal region, inferior parietal region, superior temporal region, transverse temporal region, middle temporal region, inferior temporal region, parietal white matter, temporal white matter, posterior corona radiata, and superior longitudinal fasciculus.
M3	Distal to where arteries exit the Sylvian fissure (the annotations from CTA did not specify between different M3 branches).	The individual neuroanatomical regions from the M1d downstream region.
ACA	The anterior cerebral artery.	Superior frontal region, lateral division of orbitofrontal region, medial division of orbitofrontal region, paracentral region, anterior cingulate region, posterior cingulate region, isthmus cingulate, accumbens area, and corpus callosum.
PC	The arteries of the posterior circulation i.e., the vertebral arteries, the basilar artery, and the posterior cerebral artery.	Mesencephalon, pons, medulla oblongate, vermis, precuneal region, cuneal region, pericalcarine region, lingual region, lateral occipital region, fusiform region, thalamus, cerebellum, occipital white matter, and corticospinal tract.

The regions from a neuroanatomical CT-MRI brain atlas specific to the stroke population were grouped to constitute downstream regions that correspond to vessel occlusion locations (12). We adhere to the nomenclature of the atlas to indicate the neuroanatomical regions.

###  Defining downstream regions for vessel occlusion locations

We adopted a neuroanatomical CT-MRI brain atlas specific to the stroke population from Kaffenberger et al. to define the downstream regions corresponding to the vessel occlusion locations (12). The neuroanatomical regions in the atlas were grouped to constitute these downstream regions in each hemisphere separately as shown in Table 1 (see Figure 1).

###  Determining hypoperfused regions with CTP

The CTP scans were first corrected for motion by a three-dimensional rigid registration on the skull with Elastix (13). Subsequently, the registered CTP scans were smoothed using a bilateral filter with a kernel of size 3 × 3 × 3 mm × 20 HU. The arterial input function was determined automatically as described elsewhere (14). An in-house developed model-based non-linear regression method generated the perfusion maps of the cerebral blood flow, the cerebral blood volume, the mean transit time, and the time to peak (15). From these perfusion maps, a logistic model (that is described elsewhere) determined the hypoperfused region (i.e., the infarct core and the penumbra taken together) (16).

###  Indicating vessel occlusion locations with CTP

For each patient, we aligned the atlas with the downstream regions to the CTP scan by an affine transformation using Elastix. For each downstream region, we calculated the intersection-over-union with the hypoperfused region, i.e., the volume of the region that is both the downstream region and the hypoperfused region (the intersection) divided by the volume of the region that is either the downstream region or the hypoperfused region or both (the union). The downstream region with the highest intersection-over-union with the hypoperfused region was considered to indicate the vessel occlusion location. We restricted the calculation of the intersection-over-union to slices with a perfusion deficit, and we used the Zadeh operators to determine the intersection and the union (17).

###  Assessing classification with performance metrics

We compared the indicated vessel occlusion locations from CTP and the annotated vessel occlusion locations from CTA with a confusion matrix (also known as an error matrix), considering the annotations from CTA as the reference class and the indications from CTP as the predicted class. To elicit some properties of our atlas-based model, we also looked at two (separate) variations of this confusion matrix. The first variation was to consider both the best and second-best downstream regions in order to examine the extent of wrong indications. For example, if the annotation was M1p, the best indication was M1d, and the second-best indication was M1p, then we would count the classification as correct. The second variation was a dichotomization of vessel occlusion locations into anterior large vessel occlusions and other vessel occlusions because CTP has been shown to improve the detection of CTA of vessel occlusions that are not anterior large vessel occlusions (9, 10). Anterior large vessel occlusions were defined as ICA, M1p, M1d, and the first segment of the ACA (A1). We visualized the confusion matrices with stacked bar graphs.

We derived several performance metrics from the confusion matrices. The accuracy is the number of matching annotations and indications divided by the number of patients. For each vessel occlusion location, we determined the precision and the recall. Given an indication, the precision is the probability that it matches the annotation. Given an annotation, the recall is the probability that it matches the indication.

To indicate the agreement between annotations and indications, we computed Cohen's kappa coefficient (with its 95% confidence interval). Cohen's kappa coefficient is a statistic that measures the overall agreement between two different categorizations of the same data. Mathematically, Cohen's kappa is defined as one minus the quotient of the relative observed disagreement and the hypothetical probability of chance disagreement. We evaluated Cohen's kappa coefficient qualitatively according to the levels of agreement: poor (<0.00), slight (0.00–0.20), fair (0.21–0.40), moderate (0.41–0.60), substantial (0.61–0.80), and almost perfect (0.81–1.00) (18).

## Results

A total of 620 patients were included. The included patients had a mean age of 67 years (SD: 15 years), a median NIHSS quintile of 4 (Q1–Q3: 3–5), a mean time from onset to imaging of 148 min (SD: 121 min), a median 3-month follow-up mRS of 3 (Q1–Q3: 1–4), and 57% were male (*N*: 353).

Table 2 shows the confusion matrix for the annotations and indications. The annotations and indications matched 48% (299/620) of the patients. Based on chance alone, the classification of seven categories would result in an accuracy of 14% (=1/7). For 23 patients (4%), there was no hypoperfused region.

**Table 2:** The confusion matrix for the annotated vessel occlusion locations from CT angiography (CTA) and the indicated vessel occlusion locations from CT perfusion (CTP).

	**ICA (CTA)**	**M1p (CTA)**	**M1d (CTA)**	**M2 (CTA)**	**M3 (CTA)**	**ACA (CTA)**	**PC (CTA)**
ICA (CTP)	14 [13%]	14 [19%]	7 [5%]	5 [3%]	1 [3%]	1 [7%]	1 [1%]
M1p (CTP)	25 [24%]	38 [52%]	22 [16%]	5 [3%]	1 [3%]	0 [0%]	2 [2%]
M1d (CTP)	23 [22%]	13 [18%]	82 [60%]	32 [20%]	0 [0%]	0 [0%]	0 [0%]
M2 (CTP)	22 [21%]	4 [5%]	16 [12%]	76 [47%]	13 [36%]	3 [21%]	0 [0%]
M3 (CTP)	9 [9%]	1 [1%]	4 [3%]	31 [19%]	16 [44%]	2 [14%]	8 [9%]
ACA (CTP)	1 [1%]	0 [0%]	1 [1%]	1 [1%]	1 [3%]	6 [43%]	5 [5%]
PC (CTP)	7 [7%]	1 [1%]	4 [3%]	9 [6%]	1 [3%]	2 [14%]	67 [72%]
None (CTP)	4 [4%]	2 [3%]	0 [0%]	4 [2%]	3 [8%]	0 [0%]	10 [11%]

We considered the annotations from CTA as the reference class and the indications from CTP as the predicted class. The percentages in square brackets are over the columns. The overall accuracy was 48% (299/620).

**Figure 2:**
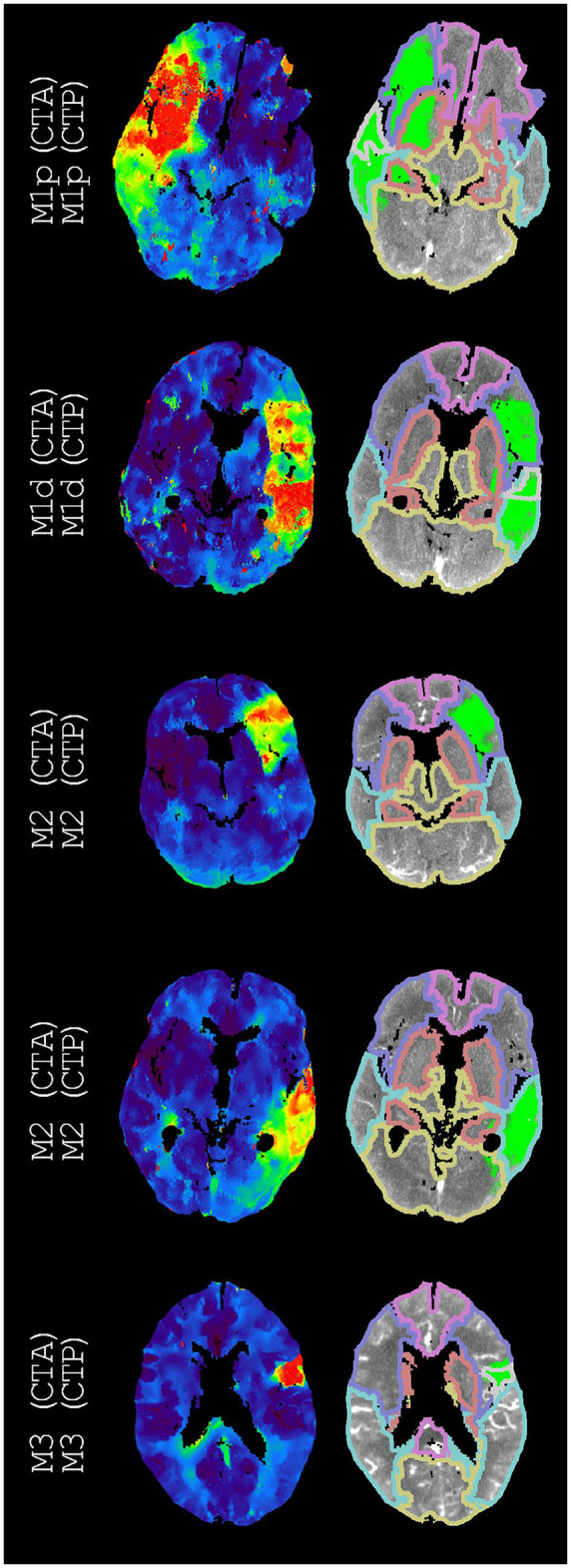
Examples of annotations (CTA) and indications (CTP) that match different segments of the middle cerebral artery. On the left, time-to-peak perfusion maps are shown. On the right, the hypoperfused regions and the downstream regions (according to Figure 1) are shown. For each example, we only regarded the M3 downstream region with the highest intersection-over-union. If displayed, these M3 downstream regions are the superior temporal region, the superior temporal region, and the precentral region (from top to bottom).

**Figure 3:**
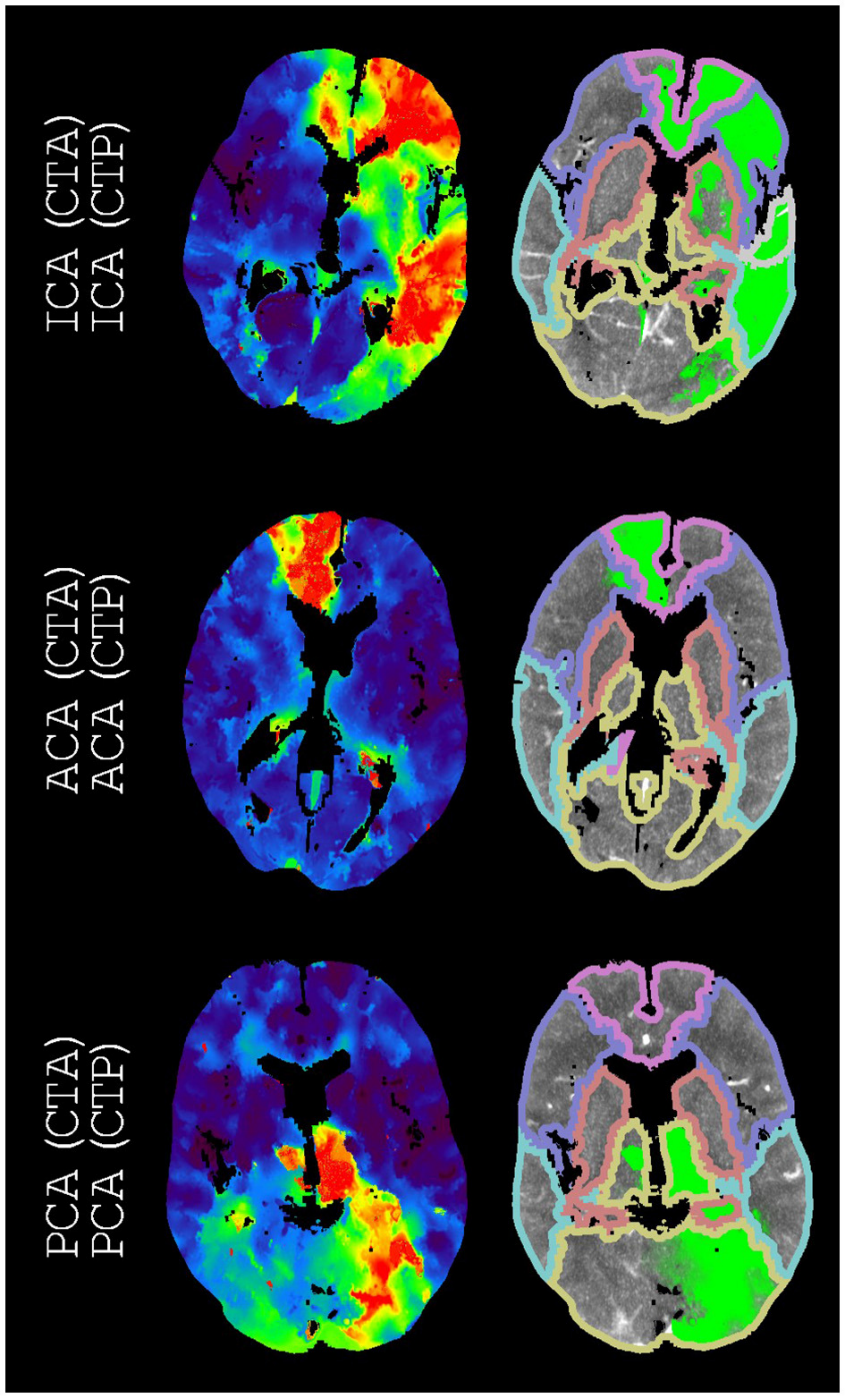
Examples of annotations (CTA) and indications (CTP) that match arteries other than the middle cerebral artery. On the left, time-to-peak perfusion maps are shown. On the right, the hypoperfused regions and the downstream regions (according to Figure 1) are shown. For each example, we only regarded the M3 downstream region with the highest intersection-over-union. The only displayed M3 downstream region is the superior temporal region.

**Figure 4:**
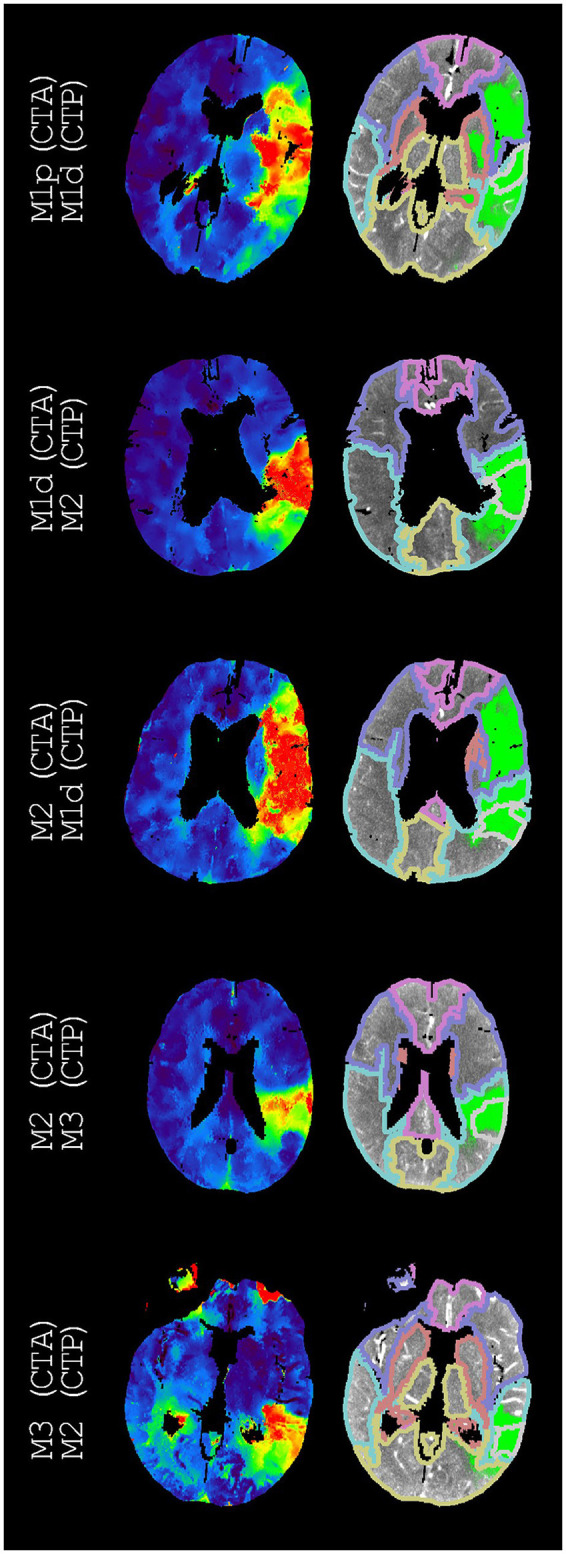
Examples of annotations (CTA) and indications (CTP) that do not match different segments of the middle cerebral artery. On the left, time-to-peak perfusion maps are shown. On the right, the hypoperfused regions and the downstream regions (according to Figure 1) are shown. For each example, we only regarded the M3 downstream region with the highest intersection-over-union. These M3 downstream regions are the superior temporal region, the supramarginal region, the supramarginal region, the supramarginal region, and the middle temporal region (from top to bottom). The second best indications were M1p, M1d, M1p, M2, and M1d (from top to bottom).

**Figure 5:**
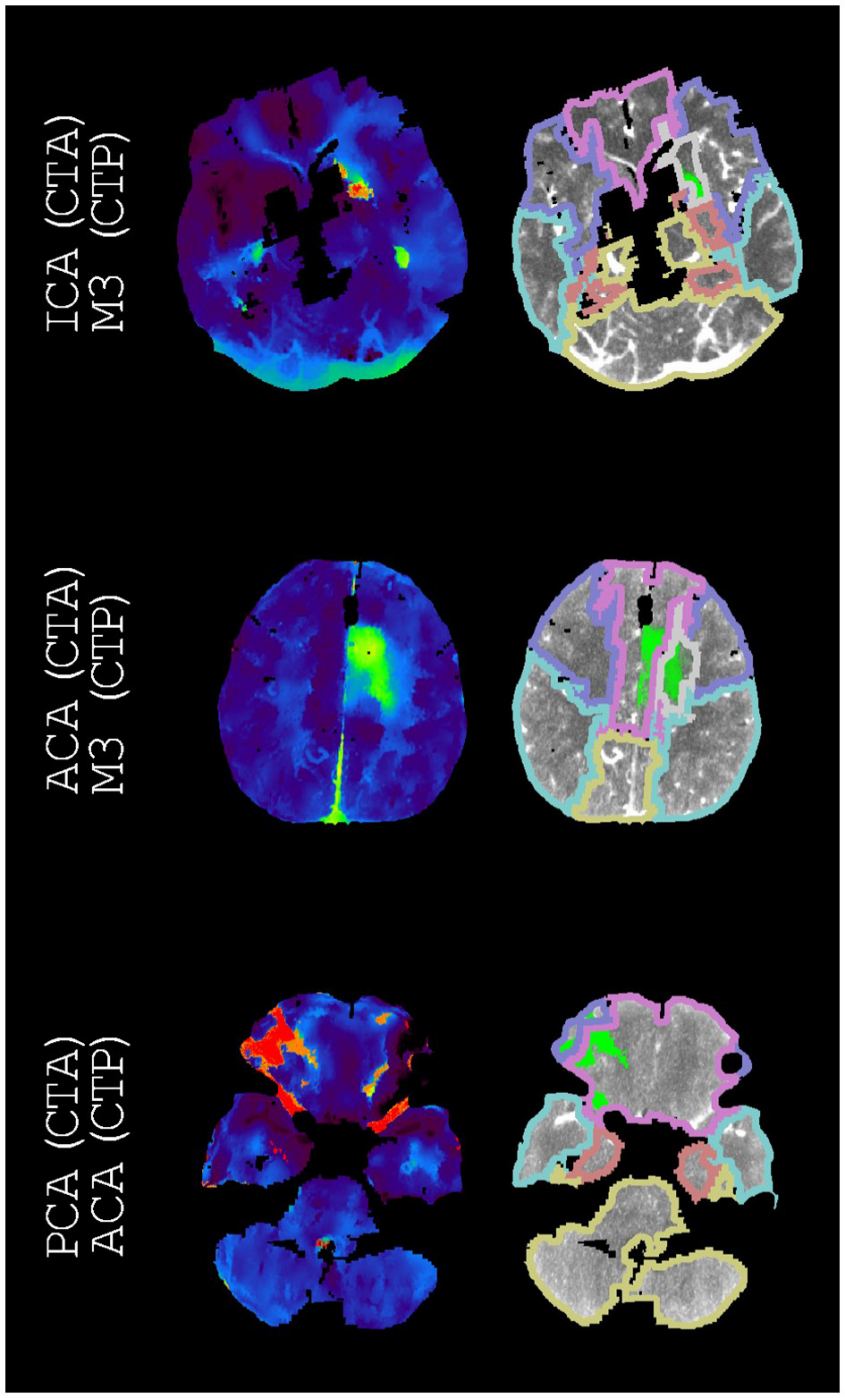
Examples of annotations (CTA) and indications (CTP) that do not match arteries other than the middle cerebral artery. On the left, time-to-peak perfusion maps are shown. On the right, the hypoperfused regions and the downstream regions (according to Figure 1) are shown. For each example, we only regarded the M3 downstream region with the highest intersection-over-union. If displayed, these M3 downstream regions are the frontal white matter and the frontal white matter (from top to bottom). The second-best indications were M2, ACA, and M2 (from top to bottom).

Table 3 presents the accuracy, Cohen's kappa, precision, and recall for each vessel occlusion location. The mean accuracy for these location-specific performance assessments was 86%, the mean precision was 46%, and the mean recall was 47%. Overall, Cohen's kappa coefficient (95% confidence interval) was 0.39 (0.35, 0.44), which indicates fair agreement. Figures 2–5 show examples of the downstream regions and the hypoperfused regions for different annotations and indications.

**Table 3:** The accuracy, Cohen's kappa, precision, and recall for each vessel occlusion location.

	**ICA**	**M1p**	**M1d**	**M2**	**M3**	**ACA**	**PC**
Accuracy	81%	85%	80%	77%	88%	97%	92%
Kappa	0.10	0.38	0.45	0.36	0.24	0.40	0.68
Precision	33%	41%	55%	57%	23%	40%	74%
Recall	13%	52%	60%	47%	44%	43%	72%

We considered the annotated vessel occlusion locations from CT angiography as the reference class and the indicated vessel occlusion locations from CT perfusion as the predicted class. The mean accuracy was 86%, the mean precision was 46%, and the mean recall was 47%.

Table 4 shows the confusion matrix for considering both the best and second-best downstream regions. The precision and the recall for the vessel occlusion locations can be found in the Supplementary material. This allowance yielded 410/620 correct matches, a mean accuracy of 91%, a mean precision of 64%, a mean recall of 64%, and an overall Cohen's kappa coefficient (95% confidence interval) of 0.62 (0.57, 0.67), which indicates substantial agreement.

**Table 4:** The confusion matrix for the annotated vessel occlusion locations from CT angiography (CTA) and the indicated vessel occlusion locations from CT perfusion (CTP) if we allow the second-best indication to also count as correct.

	**ICA (CTA)**	**M1p (CTA)**	**M1d (CTA)**	**M2 (CTA)**	**M3 (CTA)**	**ACA (CTA)**	**PC (CTA)**
ICA (CTP)	25 [24%]	3 [4%]	7 [5%]	4 [2%]	1 [3%]	1 [7%]	0 [0%]
M1p (CTP)	21 [20%]	61 [84%]	3 [2%]	5 [3%]	1 [3%]	0 [0%]	2 [2%]
M1d (CTP)	23 [22%]	3 [4%]	114 [84%]	27 [17%]	0 [0%]	0 [0%]	0 [0%]
M2 (CTP)	22 [21%]	2 [3%]	3 [2%]	110 [67%]	5 [14%]	3 [21%]	0 [0%]
M3 (CTP)	8 [8%]	1 [1%]	4 [3%]	3 [2%]	24 [67%]	1 [7%]	7 [8%]
ACA (CTP)	0 [0%]	0 [0%]	1 [1%]	1 [1%]	1 [3%]	7 [50%]	5 [5%]
PC (CTP)	2 [2%]	1 [1%]	4 [3%]	9 [6%]	1 [3%]	2 [14%]	69 [74%]
None (CTP)	4 [4%]	2 [3%]	0 [0%]	4 [2%]	3 [8%]	0 [0%]	10 [11%]

We considered the annotations from CTA as the reference class and the indications from CTP as the predicted class. The percentages in square brackets are over the columns. The overall accuracy was 66% (410/620).

Table 5 shows the confusion matrix for the dichotomization of vessel occlusion locations into anterior large vessel occlusions and other vessel occlusions. The precision and the recall for the vessel occlusion locations can be found in the Supplementary material. This dichotomization yielded an accuracy of 80% (479/597), a mean precision of 80%, a mean recall of 80%, and a Cohen's kappa coefficient (95% confidence interval) of 0.61 (0.54, 0.67), which indicates substantial agreement.

**Table 5:** The confusion matrix for the annotated vessel occlusion locations from CT angiography (CTA) and the indicated vessel occlusion locations from CT perfusion (CTP) if we dichotomize the vessel occlusion locations into anterior large vessel occlusions and other vessel occlusions.

	**Anterior large vessel occlusion (CTA)**	**Other vessel occlusion (CTA)**
Anterior large vessel occlusion (CTP)	238 [77%]	48 [17%]
Other vessel occlusion (CTP)	70 [23%]	241 [83%]

We considered the annotations from CTA as the reference class and the indications from CTP as the predicted class. The percentages in square brackets are over the columns. The overall accuracy was 80% (479/597).

**Figure 6:**
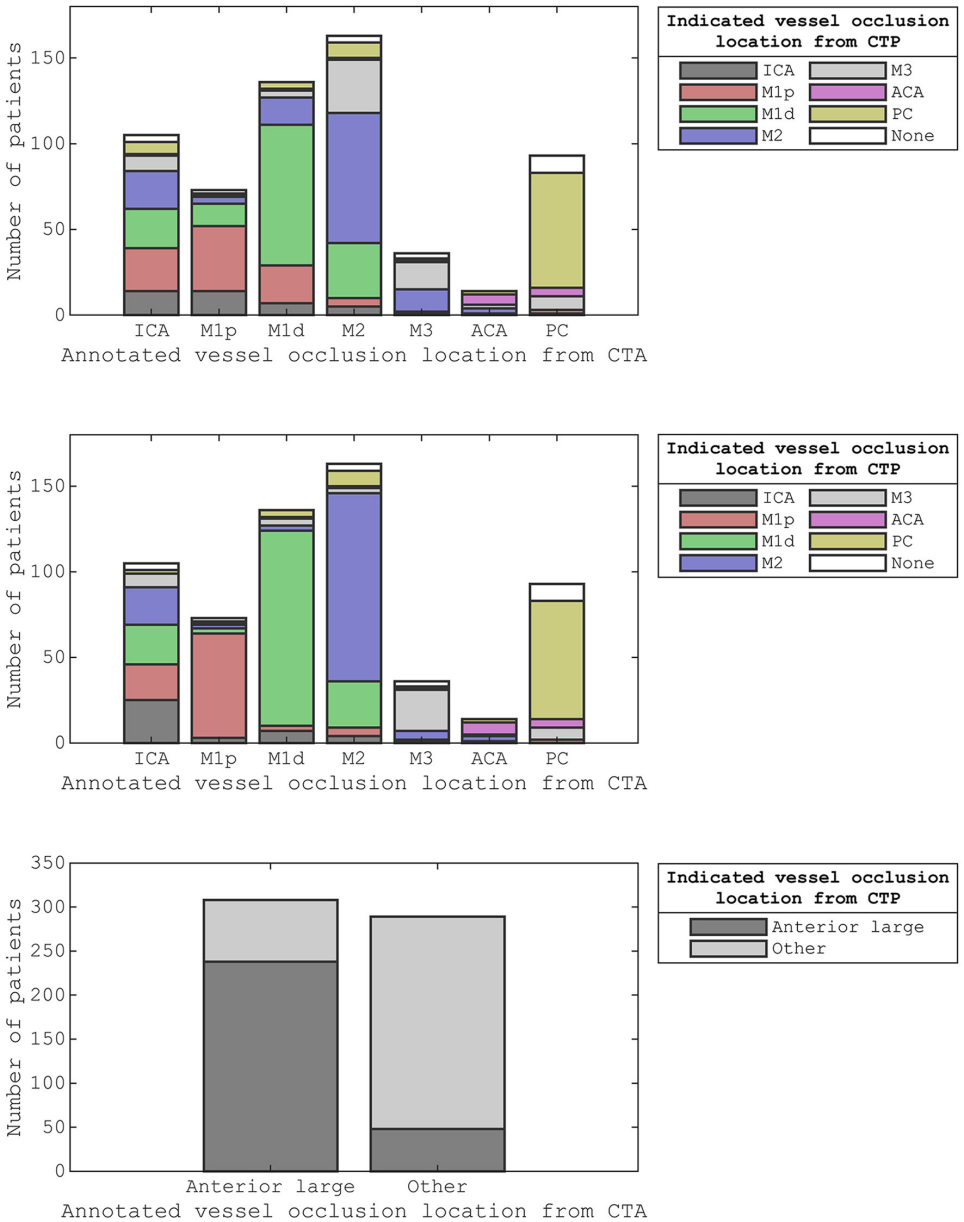
The stacked bar graphs for the confusion matrices from Table 2 (top), Table 4 (middle), and Table 5 (bottom). We considered the annotated vessel occlusion locations from CT angiography as the reference class and the indicated vessel occlusion locations from CT perfusion as the predicted class. For the middle-stacked bar graph, we allowed the second-best indication to also count as correct. For the bottom stacked bar graph, we dichotomized the vessel occlusion locations into anterior large vessel occlusions and other vessel occlusions.

Figure 6 shows the stacked bar graphs corresponding to the confusion matrices of Tables 2, 4, 5.

## Discussion

We presented an atlas-based method in which CTP data were used to automatically indicate the vessel occlusion location in acute ischemic stroke patients. We evaluated the performance of these indications by comparing them with the vessel occlusion locations acquired from expert visual assessments of CTA. The inter-rater agreement between the annotations from CTA and the indications from CTP was fair to substantial.

We focused on locating vessel occlusions instead of detecting vessel occlusions. Studies that automatically detected vessel occlusions on CTA did not evaluate their performance in locating vessel occlusions (19–24). Instead, these studies were designed to distinguish a group of patients with a large vessel occlusion from a group of patients without a detectable vessel occlusion. Similarly, experienced readers of CTP images could distinguish a group of patients with distal vessel occlusion from a group of patients without detectable vessel occlusion (5). Although these readers did not report the vessel occlusion location, their sensitivity for detecting vessel occlusions with CTP imaging agrees with the percentage of patients that did not show hypoperfusion in our study. Furthermore, expert readers of multiphase CTA images could distinguish a group of patients with a medium vessel occlusion from a group of patients without a detectable vessel occlusion, with a large vessel occlusion, or with the occlusion of the vertebrobasilar circulation (25). This last study reported that 70% (81/116) of vessel occlusions were located correctly, whereas we indicated 48% (299/620) of vessel occlusion locations correctly.

Our atlas-based method varied in performance by the vessel occlusion location (Table 3). In particular, ICA annotations had the lowest recall (14%), and ICA indications had the second lowest precision (33%). This relative underperformance may be explained by the larger potential for blood supply *via* conduit collaterals for ICA annotated occlusions than for other vessel occlusions. Conduit collaterals such as the circle of Willis can redirect blood flow based on the patient-specific angioarchitecture, resulting in hypoperfused regions that may be highly variable between patients (comparing Figures 3, 5) (26–28).

Considering both the best and second-best downstream region improved our overall performance. Although the performance for occlusions of the different segments of the middle cerebral artery improved, the performance stayed roughly equal for middle cerebral artery occlusions as a whole as well as for ICA occlusions, ACA occlusions, and PC occlusions (Figure 6). This partial improvement may be due to the variation in vessel architecture of the middle cerebral artery, resulting in a considerable number of consequential boundary cases (3). For the different segments of the middle cerebral artery, only M2 annotations with an M1d indication were not resolved by considering both the best and second-best downstream region. For these M2 annotations, the second-best downstream region often was M1p because of the relatively small difference between the M1p downstream region and the M1d downstream region (Figure 4). Thus, the performance of our atlas-based method likely suffered from variation in vessel architecture, emphasizing the need for an approach that incorporates patient-specific vessel data to locate vessel occlusions.

Our atlas-based method was limited by its design. It was unfeasible to differentiate between all possible vessel occlusion locations. To illustrate, we may consider the occlusion of the recurrent artery of Heubner, the largest perforating branch from the proximal anterior cerebral artery. Occlusion of this artery may result in a perfusion deficit that is restricted to the basal ganglia. Although this perfusion deficit is distinctly different from an M1p occlusion, our atlas-based method would indicate M1p. For the sake of clarity, we decided to only allow ordinal arteries to have overlapping downstream regions/have no overlapping downstream regions of arteries that are not in ordinal relation.

Several shortcomings should be noted. Although the defined downstream regions appear reasonable (Figures 2, 4), the performance of our atlas-based method seemed to suffer from physiological variation, for example, in collateral circulation or in M2 angioarchitecture. Furthermore, in contrast with the middle cerebral artery, we did not consider occlusions of different vessels in the posterior circulation because of a limited number of patients with occlusions in the posterior circulation. Moreover, there might be a number of patients with a missed vessel occlusion on the CTA images, especially for the smaller vessel occlusions. Unfortunately, the majority of the patients included in the DUST study did not have digital subtraction angiography (DSA) imaging, so we could not compare CTA and DSA imaging for this group. Finally, technical limitations (e.g., artifacts, the registration, or the perfusion analysis) may have resulted in an inaccurate hypoperfused region, an inaccurate downstream region, an inaccurate alignment of the hypoperfused region and the downstream region, or an unfortunate scoring by the intersection-over-union (Figures 3, 5).

In conclusion, spatial CTP data can help to automatically locate vessel occlusions for acute ischemic stroke patients. However, variations in vessel architecture between patients seemed to limit the capacity of CTP data to distinguish between vessel occlusion locations more accurately. Nevertheless, the spatial layout of the hypoperfused region might be employed in combination with patient-specific vessel data to locate vessel occlusions more effectively.

## Data availability statement

The original contributions presented in the study are included in the article/Supplementary material, further inquiries can be directed to the corresponding author.

## Ethics statement

The studies involving human participants were reviewed and approved by Institutional Review Board of University Medical Center Utrecht. The patients/participants provided their written informed consent to participate in this study. Written informed consent was obtained from the individual(s) for the publication of any potentially identifiable images or data included in this article.

## DUST (Dutch acute stroke) study investigators

The DUtch acute STroke trial (DUST) investigators are: Academic Medical Center, Amsterdam, The Netherlands (Majoie CB, Roos YB); Catharina Hospital, Eindhoven, The Netherlands (Duijm LE, Keizer K); Erasmus Medical Center, Rotterdam, The Netherlands (van der Lugt A, Dippel DW); Gelre Hospitals, Apeldoorn, The Netherlands (Droogh - de Greeve KE, Bienfait HP); Leiden University Medical Center, Leiden, The Netherlands (van Walderveen MA, Wermer MJ); Medical Center Haaglanden, The Hague, The Netherlands (Lycklama à Nijeholt GJ, Boiten J); Onze Lieve Vrouwe Gasthuis, Amsterdam, The Netherlands (Duyndam D, Kwa VI); Radboud University Nijmegen Medical Centre, Nijmegen, The Netherlands (Meijer FJ, van Dijk EJ); Rijnstate Hospital, Arnhem, The Netherlands (Kesselring FO, Hofmeijer J); St. Antonius Hospital, Nieuwegein, The Netherlands (Vos JA, Schonewille WJ); St. Elisabeth Hospital, Tilburg, The Netherlands (van Rooij WJ, de Kort PL); St. Franciscus Hospital, Rotterdam, The Netherlands (Pleiter CC, Bakker SL); University Medical Center Utrecht, Utrecht, The Netherlands (Velthuis BK, van der Schaaf IC, Dankbaar JW, Mali WP, van Seeters T, Horsch AD, Niesten JM, Biessels GJ, Kappelle LJ, Luitse MJ, van der Graaf Y); and VU Medical Center, Amsterdam, The Netherlands (Bot J, Visser MC).

## Author contributions

DP: study design, analysis, interpretation of data, and manuscript draft. HM: study concept, study design, and manuscript revision. JD and BE: study design and manuscript revision. HJ, EB, BV, and CM: manuscript revision. All authors contributed to the article and approved the submitted version.
