# Supplementary material for: Spatial CT perfusion data helpful in automatically locating vessel occlusions for acute ischemic stroke patients
Source: Front Neurol. 2023 Mar 29;14:1136232. doi: 10.3389/fneur.2023.1136232 (PMC10090274; doi:10.3389/fneur.2023.1136232)
Supplement: Supplementary file 1 [file Table_1.DOCX]

Supplementary Material

Spatial CT perfusion data helpful in automatically locating vessel occlusions for acute ischemic stroke patients

Daan Peerlings^1*^, Hugo W.A.M. de Jong^1^, Edwin Bennink^1,2^, Jan W. Dankbaar^1^, Birgitta K. Velthuis^1^, Bart J. Emmer^3^, Charles B.L.M. Majoie^3^, Henk A. Marquering^3,4^

*** Correspondence:** Daan Peerlings: d.peerlings@umcutrecht.nl

# Supplementary Tables

**Supplementary Table 1**

The precision and the recall for each vessel occlusion location if we allow the second best indication to also count as correct. We considered the annotated vessel occlusion locations from CT angiography as the reference class and the indicated vessel occlusion locations from CT perfusion as the predicted class. The mean accuracy was 91%, the mean precision was 64%, and the mean recall was 64%.

|  | ICA | M1p | M1d | M2 | M3 | ACA | PC |
| --- | --- | --- | --- | --- | --- | --- | --- |
| Accuracy | 85% | 93% | 88% | 86% | 94% | 98% | 93% |
| Kappa | 0.27 | 0.69 | 0.67 | 0.62 | 0.54 | 0.47 | 0.72 |
| Precision | 61% | 66% | 68% | 76% | 50% | 47% | 78% |
| Recall | 24% | 84% | 84% | 67% | 67% | 50% | 74% |

**Supplementary Table 2**

The precision and the recall for each vessel occlusion location if we dichotomize the vessel occlusion locations into anterior large vessel occlusions and other vessel occlusions. We considered the annotated vessel occlusion locations from CT angiography as the reference class and the indicated vessel occlusion locations from CT perfusion as the predicted class. The mean precision was 80% and the mean recall was 80%.

|  | Anterior large vessel occlusion | Other vessel occlusion |
| --- | --- | --- |
| Precision | 83% | 77% |
| Recall | 77% | 83% |
